# A Novel Comprehensive Evaluation Method for Estimating the Bank Profile Shape and Dimensions of Stable Channels Using the Maximum Entropy Principle

**DOI:** 10.3390/e22111218

**Published:** 2020-10-26

**Authors:** Hossein Bonakdari, Azadeh Gholami, Amir Mosavi, Amin Kazemian-Kale-Kale, Isa Ebtehaj, Amir Hossein Azimi

**Affiliations:** 1 Department of Soils and Agri-Food Engineering, Université Laval, Québec, QC G1V0A6, Canada; Hossein.bonakdari@fsaa.ulaval.ca (H.B.); isa.ebtehaj.1@ulaval.ca (I.E.); 2 Environmental Research Centre, Department of Civil Engineering, Razi University, Kermanshah 6714414971, Iran; Gholamiazadeh1@gmail.com (A.G.); Aminkazemi_akkk@yahoo.com (A.K.-K.-K.); 3 Faculty of Civil Engineering, Technische Universität Dresden, 01069 Dresden, Germany; 4 Institute of Research and Development, Duy Tan University, Da Nang 550000, Vietnam; 5 School of Economics and Business, Norwegian University of Life Sciences, 1430 Ås, Norway; 6 Department of Civil Engineering, Lakehead University, 955 Oliver Rd, Thunder Bay, ON P7B 5E1, Canada; azimi@lakeheadu.ca

**Keywords:** water resources, channel, mathematical entropy model, bank profile shape, gene expression programming (GEP), entropy, genetic programming, artificial intelligence, data science, big data

## Abstract

This paper presents an extensive and practical study of the estimation of stable channel bank shape and dimensions using the maximum entropy principle. The transverse slope ($S_{t}$) distribution of threshold channel bank cross-sections satisfies the properties of the probability space. The entropy of $S_{t}$ is subject to two constraint conditions, and the principle of maximum entropy must be applied to find the least biased probability distribution. Accordingly, the Lagrange multiplier (*λ*) as a critical parameter in the entropy equation is calculated numerically based on the maximum entropy principle. The main goal of the present paper is the investigation of the hydraulic parameters influence governing the mean transverse slope ($\bar{S_{t}}$) value comprehensively using a Gene Expression Programming (GEP) by knowing the initial information (discharge (*Q*) and mean sediment size (*d*_50_)) related to the intended problem. An explicit and simple equation of the $\bar{S_{t}}$ of banks and the geometric and hydraulic parameters of flow is introduced based on the GEP in combination with the previous shape profile equation related to previous researchers. Therefore, a reliable numerical hybrid model is designed, namely Entropy-based Design Model of Threshold Channels (EDMTC) based on entropy theory combined with the evolutionary algorithm of the GEP model, for estimating the bank profile shape and also dimensions of threshold channels. A wide range of laboratory and field data are utilized to verify the proposed EDMTC. The results demonstrate that the used Shannon entropy model is accurate with a lower average value of Mean Absolute Relative Error (MARE) equal to 0.317 than a previous model proposed by Cao and Knight (1997) (MARE = 0.98) in estimating the bank profile shape of threshold channels based on entropy for the first time. Furthermore, the EDMTC proposed in this paper has acceptable accuracy in predicting the shape profile and consequently, the dimensions of threshold channel banks with a wide range of laboratory and field data when only the channel hydraulic characteristics (e.g., *Q* and *d*_50_) are known. Thus, EDMTC can be used in threshold channel design and implementation applications in cases when the channel characteristics are unknown. Furthermore, the uncertainty analysis of the EDMTC supports the model’s high reliability with a Width of Uncertainty Bound (*WUB*) of ±0.03 and standard deviation ($S_{d}$) of 0.24.

## 1. Introduction

The sections and dimensions of rivers and alluvial channels change due to the constant interactions between water and sediments. River and channel plans and cross-sections undergo dimensional changes until equilibrium or stable state is attained. After equilibrium, the average dimensions of a stable cross-section do not change over time; in fact, the rate of sedimentation and erosion in a channel cross-section is theoretically in equilibrium [1,2,3]. In this case, the particles on the bed and at the channel banks are in dynamic balance. In channels with coarse particles, the movement of sediments at any location in the channel contradicts the term “channel stability” [4,5]. In this type of channel, it is not possible for sediments to move without changing the channel dimensions and width [6]. Moreover, the channel dimensions and width of water surface are only preserved (channel stability) in a state when the sediment particles on the channel bed move slightly and at the banks are in the threshold of motion [7]. In such case, one problem related to river morphology is with predicting the erosion process of river banks and profile shape formation until stable sections are achieved [8,9].

The $S_{t}$ is distributed between zero value on the channel bed and the maximum $S_{t}$ value ($S_{t}{}^{+}$) at the free water surface at the water margin. $S_{t}$ distribution is related to the lateral distance (*x*) from the channel bed (*x* = 0) to the water margin. At the water margin, *x* is named *L* which is equal to the half-width of the free water surface (*B*/2) (*L* = *B*/2). Therefore, it is worth using the entropy concept in the study of the $S_{t}$ of bank profiles because the entropy concept is based on the probability principle and its relation to a channel’s geometric parameters. Furthermore, since the $S_{t}{}^{+}$ value at the free water surface is equal to *μ* (submerged static coefficient of Coulomb friction), the $S_{t}$ of the banks is affected by the hydraulic parameters of the channel cross-sections too (including flow and sediment characteristics). The $\bar{S_{t}}$ value in channels is due to the homogeneity of $S_{t}{}^{+}$ values as a result of these conditions. Because the $\bar{S_{t}}$ value is not specified for channels (and also there is no specified relation for computing it), a uniform distribution of the transverse bank slope is assumed to obtain the $\bar{S_{t}}$ value from the ratio of the maximum flow depth at the channel centerline (*h_c_*) to the corresponding lateral distance of this depth from the central channel axis (*L*). Therefore, if the channel dimension values are not specified, the $\bar{S_{t}}$ value cannot be obtained. Therefore, a novel relationship would have existed to estimated $\bar{S_{t}}$ values based on available datasets (not only channel dimensions).

Furthermore, with the obtained entropy equation it is possible to accurately predict the $S_{t}$ of the banks depending on the correct values of the Lagrange multipliers contained in the equation. Therefore, if the entropy equation can predict the transverse bank slope correctly, multiplier *λ* should be closely related to the hydraulic and geometric parameters of the banks, which has not been investigated so far except the recent study of authors. Gholami et al. [10] analyzed the sensitivity of *λ* multiplier to different hydraulic and geometric parameters. They referred to considerable impact of the maximum slope of the bank profile and the dimensionless lateral distance of the river banks on *λ* variations. Therefore, by investigating the relationship between the entropy parameters and the hydraulic and geometric parameters of a channel, it is possible to achieve a simpler equation for the transverse bank slope distribution and thus, the bank profile shape. Based on Gholami et al.’s [10] study results, a simple relation is presented based on the maximum entropy principle to compute entropy parameter using maximum and mean values of $S_{t}$. In the Consequently, the fraction obtained with the $\bar{S_{t}}$ to $S_{t}{}^{+}$ ratio (*δ*) is evaluated and a relationship between the *δ* ratio and the entropy parameter (*K = λμ*) is presented. Moreover, a regression model based on GEP is used to create a relationship between the $\bar{S_{t}}$ of the banks and the geometric and hydraulic parameters of the flow (when the channel dimensions are unknown and only the hydraulic characteristics (e.g., *Q* and *d*_50_) are available). This relationship is combined with Vigilar and Diplas’ [11] polynomial equation to present an equation for estimating the stable free surface width based on the relationship between *δ* and *K*. The EDMTC proposed in this paper is used together with the bank profile shape equation to obtain the channel bank dimensions.

## 2. Literature Review

So far, many studies have been carried out to examine channel dimensions in dynamic equilibrium state [12,13,14,15,16,17,18,19]. However, few studies have examined the bank profile shape of threshold channels or the static equilibrium of channels. Parker [6] did extensive research in this field and justified the stable channel paradox with the nonuniform shear stress distribution on the channel bed and banks due to the longitudinal transformation of the lateral flow momentum. Parker’s model estimated the bank profile shape as a cosine curve. Later, Ikeda [20] conducted extensive laboratory studies to investigate the shape of stable channel banks. Ikeda then employed a mathematical model based on Parker’s idea and presented an exponential equation for bank profile shapes. Ikeda [20] pointed out that the most influential parameters in determining the shape of stable channels are the *Q* and *d*_50_. Diplas [21] used an analytical model with their experimental data and proposed a special case of Ikeda’s [20] equation as an exponential function for a bank profile shape. Pizzuto [22] examined the stability criterion using an analytical solution of the widening process at the free water surface. Pizzuto [22] considered the shear stress redistribution due to lateral diffusion and reported an exponential function for a bank profile after channel widening stops. Diplas and Vigilar [23] presented a numerical model to assess the difference between the shape of threshold channels and a previous conventional shape (cosine) for banks. They stated that with particles that do not move along the banks, the transverse slope of the banks should be milder, in which case a wider and deeper channel would form. Hence, they introduced a fifth-degree polynomial profile shape of stable channel banks. Vigilar and Diplas [11,24,25] provided graphs for use to predict the dimensions and profile shapes of stable channel banks with a third-degree polynomial equation. This equation can accurately predict the bank profile shape, because it is in accordance with the results obtained with the equations of several other researchers who have used various other methods [26,27]. Babaeyan [7] did an extensive laboratory study and according to their observational data introduced a hyperbolic bank profile shape. Cao and Knight [28] were the first to examine the shape of bank profiles using the entropy concept. By applying the shape equation obtained with the maximum entropy principle, they reported a parabolic equation. In solving their entropy equation, the Lagrange multiplier (*λ*) contained within were tested numerically. The equation was validated according to Chow’s [29] definition of natural rivers considering a value of zero for *λ*. Cao and Knight [28] emphasized the need to further consider the physical concept of multiplier *λ*. Following Cao and Knight’s [28] brief study, no other study has been based on the entropy concept to predict the $S_{t}$ and hence the bank profile shape of stable channels. Gholami et al. [30,31,32,33,34] assessed the ability of different artificial intelligence (AI) methods in the estimation of bank profile shapes of threshold channels. They referred to high efficiency in these methods in estimation and the necessity of further researches about on forming stable shape of bank profiles.

Due to the significance of the entropy concept, many studies have addressed entropy in examining different variables [35,36,37,38]. In hydraulic science, Chiu [39] was the first to examine the flow velocity distribution using entropy. Later, other considerations were applied to evaluate the mean and maximum velocity ratio, shear stress and sediment concentration distributions in the cross sections of channels [40,41,42,43,44,45,46,47,48,49,50,51,52,53]. In the field of application of entropy concepts in determining $S_{t}$ of stable channels, recently, Gholami et al. [54,55] assessed the ability of Tsallis and Shannon entropy concepts in estimation of $S_{t}$ of stable channels banks. They extensively assessed the variation of different entropy parameters and their signs in obtained entropy-based equations. However, they presented no reports about the significant effects of relations of maximum and mean values of $S_{t}$ with entropy parameters and the other hydraulic and geometric conditions.

## 3. Materials and Methods

### 3.1. Maximum Entropy Principle in Estimating the Transverse Slope of Stable Banks

**Figure 1:**
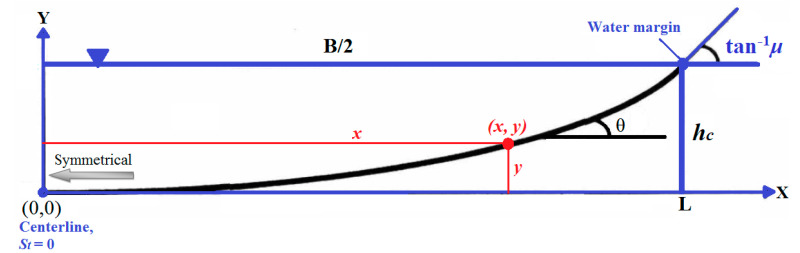
Symmetrical cross section of alluvial threshold channels and its characteristics.

Cao and Knight [28] evaluated the $S_{t}$ of banks in threshold state using the principle of maximum entropy for the first time. In the following, Gholami et al. [54,56] modified the application of maximum entropy principle used by Cao and Knight [28]. Cao and Knight [28] employed the Shannon entropy [56] in the form of Equation (1) and presented Equation (2) considering the $S_{t}$ of stable banks as a random variable and the principle of maximum entropy [57,58] associated with the two constraint conditions of continuity and momentum in Equations (3) and (4) [59].

(1) $$H(S_{t})=\;-\;\int p(S_{t})\ln p(S_{t})dS_{t},$$

where $p(S_{t})$ is the Probability Density Function (PDF) of the $S_{t}$ of the banks, and *H* is the amount of entropy.

(2) $$S_{t}=\;\;\frac{1}{\lambda }\ln\left[1+(e^{\lambda \mu }-1)\frac{x}{L}\right]$$

(3) $$\int_{0}^{\mu }p(S_{t})dS_{t}=\;1,$$

(4) $$\int_{0}^{\mu }S_{t}p(S_{t})dS_{t}=\;\bar{S_{t}},$$

where *x* is the lateral distance of points on the banks from the channel centerline and *λ* is the Lagrange multiplier. Figure 1 represents a symmetrical bank cross section of stable alluvial channels. In stable channels, $S_{t}$ of the banks changes monotonically from the centerline of the channel bed (*x* = 0 and *y* = 0) that is zero ($S_{t}$ = 0) to the $S_{t}{}^{+}$ value at the free water surface at the water margin (*x* = *L* = *B*/2 and *y* = *h_c_*), which is equal to *μ* (the submerged static coefficient of Coulomb friction).

Cao and Knight [28] carried out numerical testing and considered a specified range for *λ* (1, 5, 10, 50, 100). They stated that when *λ* tends toward zero, the cross-sectional bank shape is a parabolic curve. Consequently, this multiplier was deleted from their equation. The following equation was presented with numerical justification for bank profile shape estimation:

(5) $$y*=\left(\frac{\mu^{2}}{4}\right)\;x{*}^{2},$$

where x* = *x*/*h_c_* is the dimensionless lateral distance from the channel centerline and y* = *y*/*h_c_* is the dimensionless vertical boundary level. The Lagrange multiplier is a key component of the maximum entropy principle. In the following, Gholami et al. [54] presented an equation based on the maximum entropy principle to caculate *λ* numerically [54] which is explained in summary in the following. Accordingly, by using the Lagrange Multiplier Method (LMM) and variable calculation technique [39,60,61], the equation below is obtained for $p(S_{t})$:

(6) $$p(S_{t})=\exp(\lambda_{1}+\lambda S_{t}-1).$$

Equation (6) is used with the first constraint (Equation (3)) to obtain the following equation:

(7) $$e^{\lambda_{1}-1}=\;\lambda {\left(e^{\lambda \mu }-1\right)}^{-1},$$

where *λ*_1_ is Lagrange multipliuer and equal to: *λ*_1_ = ln[*λ*/(*e^λμ^* − 1)] + 1.

Furthermore, by replacing Equations (6) and (7) in the second constraint condition (Equation (4)), the following equation is obtained to calculate *λ*:

(8) $$\bar{S_{t}}=\frac{\mu e^{\lambda \mu }}{\left(e^{\lambda \mu }-1\right)}-\frac{1}{\lambda }.$$

On the other hand, by dividing the sides of Equation (8) by *μ*, the following equation is obtained:

(9) $$\frac{\bar{S_{t}}}{\mu }=\;\delta =\frac{e^{K}}{\left(e^{K}-1\right)}-\frac{1}{K}$$

where *K* is a dimensionless parameter known as the entropy parameter used to measure the uniformity of the probability and distribution of the $S_{t}$, which is equal to *K* = *λμ*, and *δ* is the ratio of $\bar{S_{t}}$ to $S_{t}{}^{+}$ (=*μ*). In the present study, when the values of *h_c_*, *L*, and $S_{t}{}^{+}$ (=*μ*) are known, the $\bar{S_{t}}$ value along the banks is obtained by assuming the uniform distribution of $S_{t}$ as equal to the *h_c_*/*L* ratio. Therefore, *λ* is obtained by numerically solving Equation (8). Then, the $S_{t}$ distribution of stable banks can be computed according to Equation (2). Moreover, physical justifications of *λ* multiplier and the effect of different hydraulic and geometric parameters on it is investigated in Gholami et al. [10]. On the other hand, the $S_{t}$ at each point on the channel banks is formulated as $S_{t}=dy/dx$, where *y* is the vertical boundary level of the points. By integrating this, the bank profile shape equation for threshold channels becomes Equation (10), where the integral constant (*C*) is obtained by applying the boundary condition at the channel centerline (*x* and *y* = 0).

(10) $$y=\;\frac{1}{\lambda }\left[\left(x+\frac{L}{e^{\lambda \mu }-1}\right)\ln\left(1+(e^{\lambda \mu }-1)\frac{x}{L}\right)-x\right].$$

This is introduced as the bank profile shape equation based on developed entropy model which is extended in Gholami et al. [54] in details. If the channel dimensions (*B* and *h_c_*) are not specified, it is not possible to estimate *λ* and hence, the $S_{t}$ and *y* values. Therefore, in this paper, the next section presents a numerical model for when the channel dimensions are not specified and only *Q* and *d*_50_ are known from the problem condition.

### 3.2. Calculating μ

The *μ* value can be calculated as *μ* = tan *φ*, where *φ* is the angle of sediment reposition. Furthermore, since the value of *μ* changes with the sand size and roughness [5,62], the following relationship between the *φ* and sediment size (*d*_50_) can be utilized in the current study to compute *φ* in uniform sediments [10,27,54]:

(11) $$\varphi =\left[\begin{array}{l} 0.302{\left(\log d_{50}\right)}^{5}+0.126{\left(\log d_{50}\right)}^{4}-1.811{\left(\log d_{50}\right)}^{3}\\ -0.57{\left(\log d_{50}\right)}^{2}+5.952\left(\log d_{50}\right)+37.52 \end{array}\right]$$

where *φ* is in degree and *d*_50_ should be inserted in centimeters.

### 3.3. Entropy-Based Design Model of Threshold Channels (EDMTC)

As stated in the previous section, by assuming a uniform distribution for $S_{t}$ value, the $\bar{S_{t}}$ value can be obtained by the *h_c_*/*L* when the values of *h_c_*, and *L* (=*B*/2) are known. Accordingly, if the *h_c_* and *B* values are not known, it is not possible to calculate $\bar{S_{t}}$. In this section, an explicit relationship will be provided to calculate the $\bar{S_{t}}$ value for the cases that the channel dimensions (*h_c_*, *B*) are not available.

**Figure 2:**
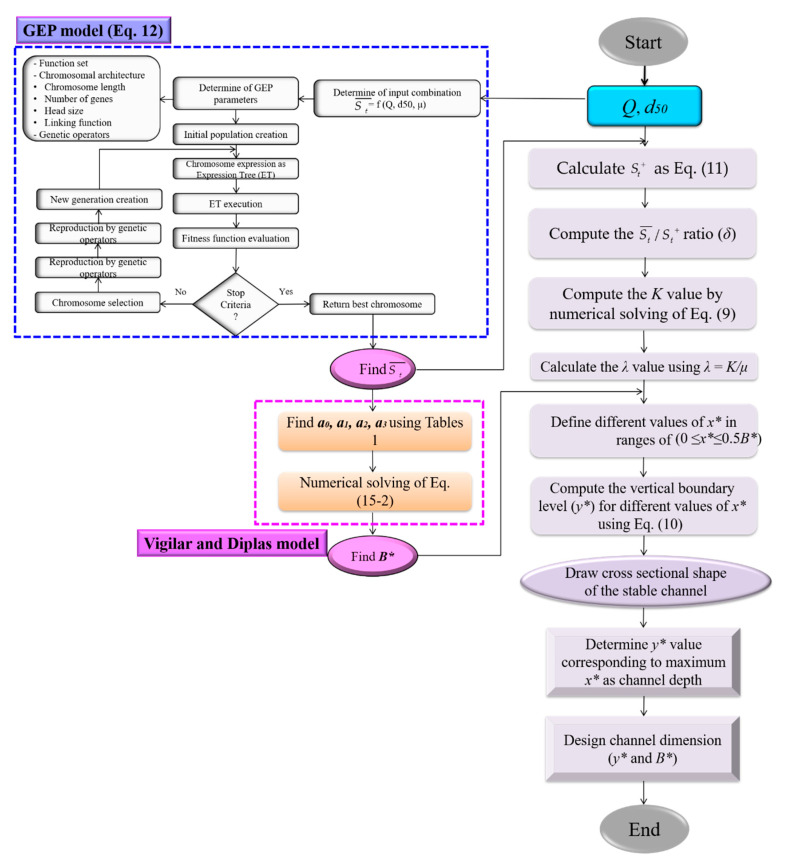
Flowchart of the proposed Entropy-based Design Model of Threshold Channels (EDMTC) computational procedure for designing the dimensions and shape of threshold channels in the present study.

In this way, using several series of available observational data with different hydraulic conditions, the *Q*, *d*_50_ and *μ* values are determined and a relationship for the $\bar{S_{t}}$ value based on these parameters is applied to calculate the $\bar{S_{t}}$ value for any other data where the channel dimensions are not specified. Accordingly, considering *Q*, *d*_50_ and *μ* parameters as input parameters and $\bar{S_{t}}$ as output parameter based on a numerical GEP model (Figure 2) [32,63,64] provide a relationship for predicting $\bar{S_{t}}$ in the form of Equation (12):

(12) St¯ = G1 + G2 + G3,G1 = e^{−{[μ2 − 2μ +ln(μ+4.433)]+[exp(−(Q+μ)2)]+exp[−((0.936+d50)2)]2}},G2 = e^{−{[(17.693−1.565Q)+(1/d50)]+[μ+1.565−μQ]}2},G3 = e^{−{[(1.112Qd50−ln(6.5Q))/μ]+μ}2}.

In fact, with input parameters *Q*, *d*_50_ and *μ* (=$S_{t}{}^{+}$) the value of $\bar{S_{t}}$ is calculated using Equation (12). Now by knowing the $\bar{S_{t}}$ value for any channel whose stability dimensions are not specified, in addition to bank profile shape, the width and depth of the channel after stability can be determined. To do this, $\bar{S_{t}}$ can be calculated by using the equations presented by former researchers who have applied analytical and theoretical frameworks to derive the relationships. As stated, the polynomial shape proposed by some researchers is an acceptable shape than the previous classic cosine, parabolic, and exponential forms [23]. Therefore, in the present study, the polynomial function provided by Vigilar and Diplas [11] is used to estimate the bank profile shape of stable channels as follows [11]:

(13) $$y*=\;1-a_{3}x{*}^{3}-a_{2}x{*}^{2}-a_{1}x*-a_{0}.$$

Coefficients *a*_0_, *a*_1_, *a*_2_ and *a*_3_ depend on the values of δ*_cr_ and *μ*, which are obtained from Table 1 for each given dataset [11]. δ*_cr_ is the dimensionless critical stress depth (δ*_cr_ = δ_cr_/h_c_) in critical condition of sediments in the bank profile. In this case, the shear stress depth (*δ′*) is *δ′ = τ/ρgS*, where *τ* is the shear stress along the channel and *S* is the longitudinal slope of the water surface. The value of δ*_cr_ can be obtained according to the (*μ* − δ*_cr_) figures related to Vigilar and Diplas [11].

Now, the derivative of the above function (Equation (13)) versus dx* yields the transverse slope function at different points in the channel as follows:

(14) $$S_{t}=\;\frac{dy*}{dx*}=\;-3a_{3}x{*}^{2}-2a_{2}x*-a_{1}.$$

Now, according to the mean value theorem in integral, the mean slope value of the bank profiles ($\bar{S_{t}}$) is computed based on the mean value theorem for definite integrals for y* distribution (Equation (13)) along the transverse interval in range of (0≤x*≤0.5B*) according to the following Equations (15a–c):

(15a) $$\bar{S_{t}}=\;\frac{1}{0.5B*}\;\int_{0}^{0.5B*}y*(x)dx,$$

(15b) $$\bar{S_{t}}=\;\frac{2}{B*}\left[1-a_{3}\frac{B{*}^{3}}{8}-a_{2}\frac{B{*}^{2}}{4}-a_{1}\frac{B*}{2}-a_{0}\right],$$

(15c) $$\bar{S_{t}}=\;-a_{3}\frac{B{*}^{2}}{4}-a_{2}\frac{B*}{2}-a_{1}-\frac{2}{B*}(a_{0}-1).$$

Therefore, by obtaining $\bar{S_{t}}$ value using Equation (12), B* value of the free water surface of bank profile is obtained with Equation (15b). In fact, with input parameters *Q*, *d*_50_ and *μ* (=$S_{t}{}^{+}$) the value of $\bar{S_{t}}$ is calculated using Equation (12). Then, Equation (15c) is used to obtain the value of B* based on obtained $\bar{S_{t}}$ values according to Equation (12). Accordingly, in this study, the EDMTC (Figure 2) is presented to predict the dimensions and shape of bank profiles using the entropy principle. The value of x* (lateral distance from the channel axis) is selected for a specific range of arbitrary x* values at a distance of $0\leq x{*}_{i}\leq 0.5B*(=L)$. The values of y* obtained by the entropy facilitate plotting the bank shape profiles against different *x_i_*. Figure 2 shows the flowchart of the GEP model and model developed in the present study (EDMTC) to predict the shape and dimensions of threshold channels.

### 3.4. Experimental Data

The observational data series used in the present study were collected in previous investigations by Mikhailova et al. [65], Ikeda [20], Diplas [19], Babaeyan [7], Macky [66], Hassanzadeh et al. [67], and Khodashenas [68]. The hydraulic and geometric conditions of the data vary, with different ranges of *Q* and *d*_50_ values in the channel as well as geometric conditions of the laboratory flumes used with each data series. Furthermore, several tests were carried out for different discharge rates with each data series, and the channels had different conditions until reaching equilibrium state. In each observational data series, in addition to the channel dimensions (*B* and *h_c_*) the coordinate data of the points in stable bank profiles (*x, y*) were extracted for some discharge values as well. Moreover, all experiments were done in laboratory flumes with different aspect ratios (*B*/*h_c_* = *α*) in the range (4–30). In each test, the sediment sizes selected were somewhat course, so the corresponding proportional discharge in the channels would cause no movement of sediment particles in the channels. Hence, the stresses on the walls and channel bed were respectively less and more than the critical stress until threshold channel conditions would govern. Table 2 summarizes the hydraulic and geometric conditions for the data used.

### 3.5. Used Data in Modeling

As stated in the previous section, in this paper, 12 numbers of observed runs (S1–S12) (according to Table 1) with different hydraulic and geometry characteristics are selected for training and testing the EDMTC model. The hydraulic and geometric conditions of the data series are varied, so that the range of *Q* and *d*_50_ values in the channel, as well as the geometric conditions of the laboratory flumes used in each data series, are different. Furthermore, in each seven available observational data series (Mikhailova et al. 1980; Ikeda 1981; Diplas 1990; Babaeyan 1996; Macky 1999; Hassanzadeh et al. 2014; and Khodashenas 2016), there are several runs related to them according below with different discharges, therefore, the stable channel shape formed on banks in each observed run is different.

- Ikeda (1981) → one run as **S3** (8 samples)
- Diplas (1990) → one run as **S4** (25 samples)
- Babaeyan (1996) → one run as **S5** (8 samples)
- Macky (1999) → one run as **S6** (101 samples)
- Hassanzadeh et al. (2014) → two runs as **S7** (33 samples) and **S8** (38 samples)
- and Khodashenas (2016) → four runs as **S9** (44 samples), **S10** (33 samples), **S11** (57 samples) and **S12** (20 samples)

In fact, in this paper, external-validation is performed. External validation means that among 12 numbers of data series (totally 367 sample numbers), some data series are used for training and some data series are selected for testing the models. Accordingly, in this paper, 10 data series of S1, S2, S3, S7, S8, S9, S10, S11, and S12 (65% of all samples: 233 samples) are used for training the EDMTC model and three data series of S4, S5, and S6 (35% of all samples: 134 samples) related to Diplas’ (1990), Babaeyan’s (1996) and Macky’s (1999) data series are selected for testing the EDMTC model. This kind of validation is acceptable, because the proposed EDMTC model is trained and tested based on data series with different hydraulic and geometry characteristics.

### 3.6. Evaluation of Model Efficiency

In order to evaluate the methods presented in this study, several statistical indices are used: The determination coefficient (*R*^2^), Root Mean Squared Error (RMSE), Mean Absolute Relative Error (MARE), Mean Absolute Error (MAE), and Bias. These evaluation criteria are defined by Equations (16)–(20):

(16) $$R^{2}=\;1-\frac{\sum_{i=1}^{n}{\left(y_{i}-x_{i}\right)}^{2}}{\sum_{i=1}^{n}{\left(y_{i}-\bar{y}\right)}^{2}},$$

(17) $$RMSE=\sqrt{\frac{1}{n}\sum_{i=1}^{n}{\left(x_{i}-y_{i}\right)}^{2}},$$

(18) $$MARE=\frac{1}{n}\sum_{i=1}^{n}\left(\frac{\left|x_{i}-y_{i}\right|}{x_{i}}\right),$$

(19) $$MAE=\frac{1}{n}\sum_{i=1}^{n}\left|x_{i}-y_{i}\right|,$$

(20) $$Bias=\frac{1}{n}\sum_{i=1}^{n}(x_{i}-y_{i}),$$

where *y_i_* and *x_i_* denote the estimated and observed values, $\bar{y}$ represents the mean modeled values and *n* is the sample size. The closer the *R*^2^ coefficient is to the unit value (1), the higher the agreement there is between the observed and predicted values. The closer the results of MARE, RMSE, Bias, and MAE indices are to zero, the higher the estimation accuracy is as well. Positive and negative Bias values imply model over and underestimation, respectively [69,70,71]. Therefore, computing several evaluation criteria can better reveal the model performance [72,73].

## 4. Results

In the first section, the ability of entropy model is evaluated to predict bank profile shapes. In the second section, the EDMTC proposed in this study is examined in detail. At the end, the uncertainty of the proposed EDMTC is examined using different uncertainty indexes.

### 4.1. Entropy Model in Predicting Bank Profile Shapes

**Figure 3:**
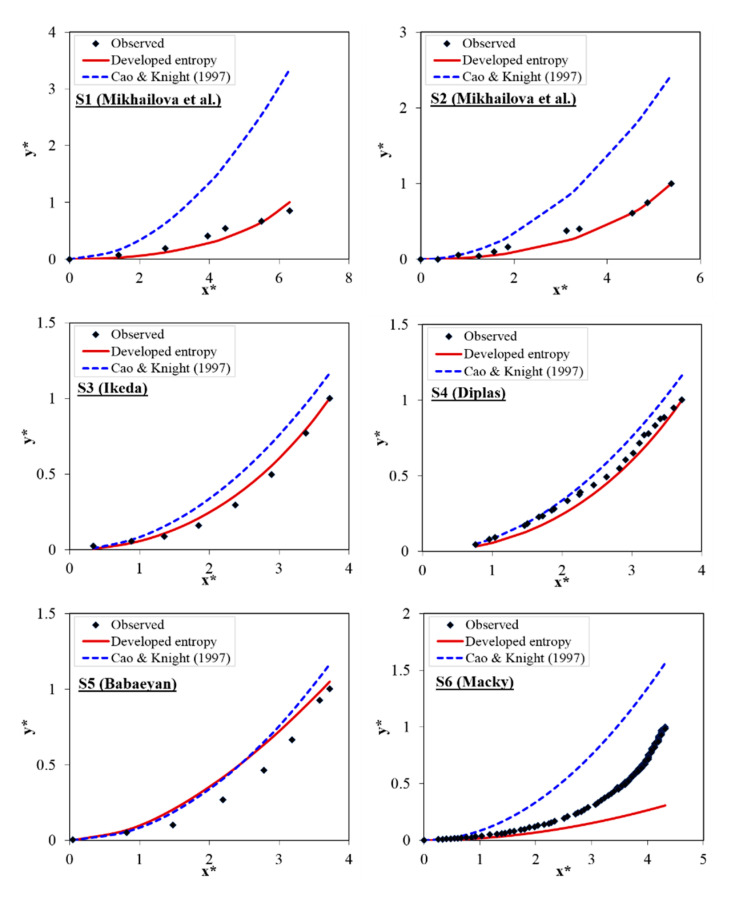

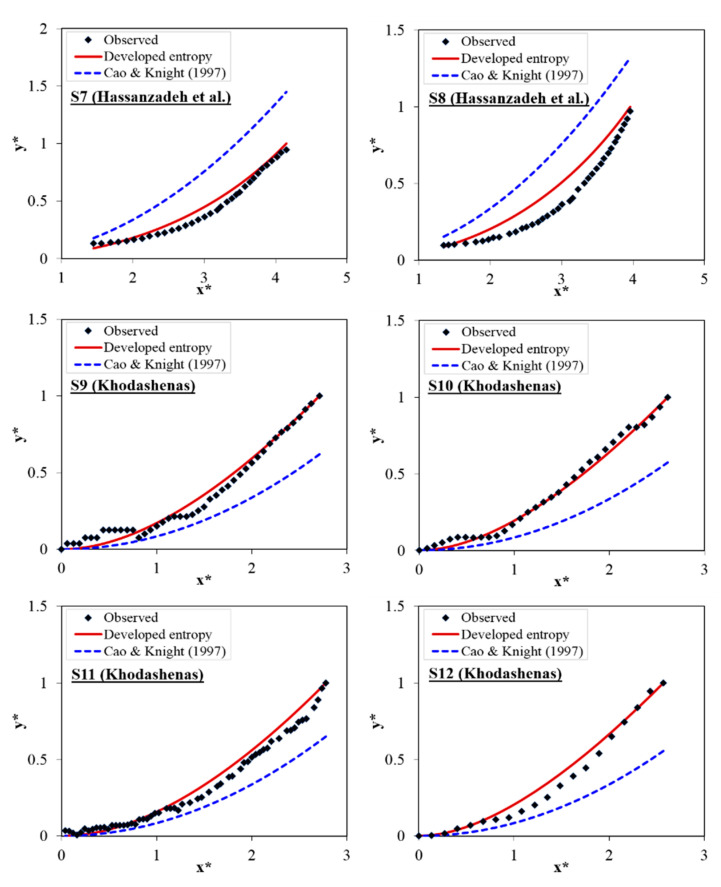
Bank profile shape predicted by developed entropy model and Cao and Knight’s [28] model (CKM) for different observational data series (S1–S12).

In Figure 3, the vertical boundary level of stable channel banks is estimated by the developed entropy model based on the maximum entropy principle which is proposed in Gholami et al. [54] for the first time. The *λ* value is obtained by numerical solution of Equation (8). Accordingly, for each data series (each bank profile shape), one *λ* value is obtained by numerically solving Equation (8). In Equation (8), $\bar{S_{t}}$ value is calculated by assuming uniform distribution of $S_{t}$, according to ratio of *h_c_*/*L*. Using obtained *λ* value, the *y* value is computed based on entropy method by solving Equation (10). The y* distribution obtained by Equation (10) corresponding each x* value is drawn for each data series in Figure 3. Moreover, the results of Cao and Knight’s [28] model (CKM) (according to Equation (5)) are extracted and their proposed bank profile shape is drawn in Figure 3 to evaluate the entropy model performance. Table 3 contains the different error indices for entropy model and CKM. Figure 3 indicates that entropy model exhibits acceptable conformity with the corresponding observational data series in predicting the vertical boundary level and hence, estimates the bank profile shape with low error values. According to all data series, entropy model is able to estimate the governing bank profile shape trend with lower MARE and RMSE values equal to 0.317 and 0.08 better than CKM with 0.981 and 0.363 values respectively. Figure 3 also shows that for two data series, i.e., S1 and S2 (Mikhailova et al.’s [65] data), CKM has high error values in y* estimation and high accuracy in the area near the free water surface, where high MARE values in the 2–4 range are observed for these data series. However, the proposed entropy model is able to detect the bank profile shape trend with lower error values (MARE = 0.2 and 0.8 for S1 and S2 datasets respectively) than CKM with 1.95 and 3.95 MARE values, which represents the significant superiority of entropy model. This process is repeated for the S2 and S3 data series. Although CKM exhibits acceptable performance, entropy model is more accurate with lower error values and coincides closely with the observed values (especially in the area near the surface). For the S6 field data series, although both models do not perform well (with close Bias values of −0.31 and 0.45 for entropy model and CKM respectively), entropy model again performs with lower error (MARE = 0.58) than CKM (MARE = 1.03). Furthermore, the high MARE index value for CKM is representative of its inability to estimate low y* values (in the vicinity of channel bed), a problem that is solved by entropy model significantly. Furthermore, the RMSE values of CKM and entropy model which is equal to 0.5 and 0.38 respectively approved the inefficiency of CKM in estimating low y* levels. With data series S7, the improvement of entropy model over CKM by about 60% and 85% in the MARE and RMSE values respectively is observed clearly in Figure 3, as entropy model highly conforms to the observational data with *R*^2^ values of 0.98. With Khodashenas’ [68] data (S9–S12), the higher efficiency of entropy model over CKM is evident with lower MARE and RMSE values in entropy model than CKM. Furthermore, entropy model is able to estimate the water surface widening with high y* values well with low values of RMSE and Bias values close to 0. The negative and positive Bias value represents the underestimation and overestimation of the models respectively. As it can be seen in the Bias values, the CKM in most of the datasets have positive Bias values and overestimates the y* values in comparison with the corresponding observed values. It can thus be said that the entropy model proposed in the present study based on the maximum entropy principle is more accurate in the estimating the bank profile shape of stable channels than CKM, which suggests a parabolic curve (Equation (5)) for channel banks. A notable point in this paper is the significant physical effect of *λ* values on the accurate estimation of the intended variables, which is negligible with CKM. The *λ* values obtained by entropy model in this study are gathered in Table 3, where it can be seen that this multiplier is in a specified range of −2 to 2 with almost all data series (except with 1–2 data series). Furthermore, the *λ* values are the same for different runs of one experiment.

### 4.2. Presenting the Entropy-Based Design Model of Threshold Channels (EDMTC)

In previous sections, the entropy model was evaluated for its prediction ability of bank profile shapes in case the depth and width of the free water surface in the channel are determined. In this study, EDMTC based on the relationship between the entropy parameter and the $S_{t}$ of channel banks to predict the channel dimensions as well as the bank profile shape is presented and explained in detail in Section 3.2 and Figure 2. The proposed EDMTC is evaluated in the first subsequent section and the model’s uncertainty is examined in the second part.

#### Evaluation of EDMTC Performance

**Figure 4:**
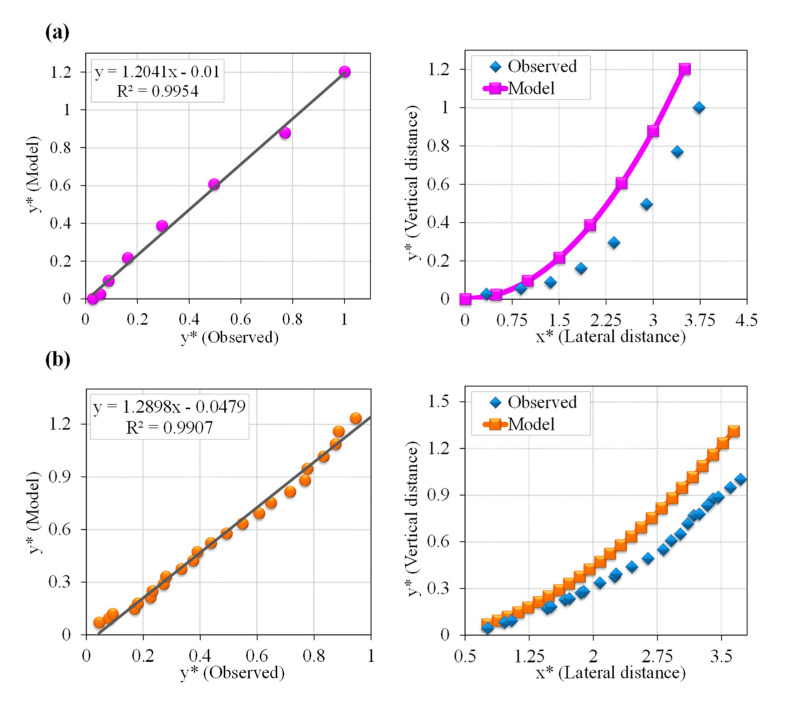

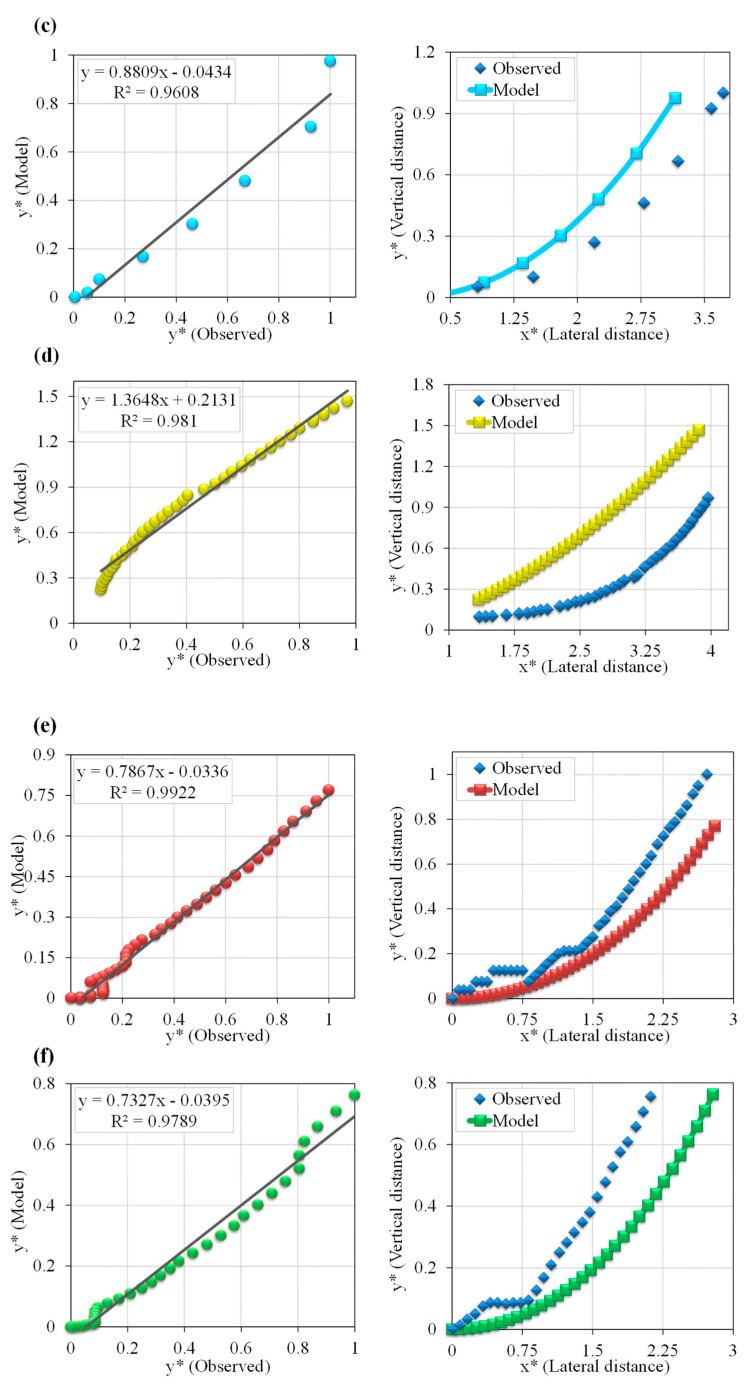
Comparison of values predicted for the vertical boundary level of stable channels by the EDMTC proposed in the present study using scatter plots (left side) and cross-sectional profile shapes (right side) for different observational data: (**a**) Ikeda [20]-S3, (**b**) Babaeyan [7]-S5, (**c**) Diplas [21]-S4, (**d**) Hassanzadeh et al. [67]-S7, (**e**) Khodashenas [68]-S9, and (**f**) Khodashenas [68]-S12.

Figure 4 displays scatter plots of the EDMTC proposed in this study for several observational data series. The left side of the figure contains the regression plots of the y* values predicted by EDMTC compared to the corresponding observational values. The right side of the figure shows the cross-sectional profile shapes predicted by EDMTC compared with the profile shapes obtained with observational values. Table 4 lists the error indices of EDMTC compared to the corresponding observational values. The scatter plots indicate that EDMTC can very accurately predict the vertical elevation of stable channel banks, as most data is compressed around the trend line and slight scattering is observed for some of the datasets. In Figure 4, the trend line is mapped to the data and the resulting equation is *y = ax + b*. Closer *a* and *b* values to 1 and 0, respectively, represent acceptable model prediction performance. According to the trend line, for all datasets the predicted values are concentrated around this line and the values of *a*, *b* are close to 1, 0, respectively. This indicates the high efficiency of the proposed EDMTC in predicting the vertical elevation of channel banks. Moreover, the *R*^2^ index value in this figure is higher than 0.95 for all observational data series, indicating the high EDMTC prediction accuracy. The value of this index is very close to 1 for some of the observational data [20,21,68], signifying very high model conformity to the corresponding observational values. Furthermore, according to the diagrams on the right side of Figure 4, the EDMTC is able to accurately estimate the bank profile shape trend for all data series. Although some differences between the values y* predicted by the model and the observational values are seen, it is notable that EDMTC is able to model the vertical bank elevation (from the channel center on the bed to the free water surface margins) and the water surface widening near the water surface levels similar to the corresponding observational values. The error index values in Table 4 are also validated accordingly. This table shows that the MARE values for all datasets are 0.3–0.5, which is close to 0. This index indicates the accuracy of the proposed EDMTC in predicting the vertical elevation of banks as well as the free water surface width in stable channels. An important point is that the proposed EDMTC predicts the profile shape trend successfully and can therefore be used to design the width and depth (dimensions) of stable channels when only flow inputs such as *Q*, *d*_50_ and *μ* are known. The high accuracy of this model is confirmed, and achieving such a model with the least parameters to predict the dimensions and cross-sectional bank shapes formed in stable channels is of considerable importance. Also, EDMTC not only considers the geometric conditions of the channel cross sections but also involves the hydraulic conditions of the problem (by using Vigilar and Diplas’ [11] equation), which is one of the notable features of this model. Based on most observational data series, the estimated channel width is very similar to the observational values (in some cases it is slightly less). For example, for the EDMTC profile predictions based on the observational data from Diplas [21], Babaeyan [7], and Hassanzadeh et al. [67], the water surface width is estimated very close to the observed values. Furthermore, for most observational datasets, the proposed model estimates greater values for the vertical elevation of the water surface, although the estimated profile trend fits the observational values perfectly. The partial error values of EDMTC that are mostly seen in the areas near the channel bed and the free water surface with some of the datasets can be considered measurement errors of the observational data [74]. For some data, e.g., Hassanzadeh et al. [67] and Khodashenas [68] this error is seen at the channel bed. Additionally, Figure 4 shows that EDMTC based on Khodashenas’ [68] data estimates lower y* than the actual values, which results in a negative Bias and an absolute error increase of 14% in MAE value according Table 4 (MAE represents the absolute magnitude of the difference between observational values and the model). It is worth noting that the EDMTC can estimate a more logical shape than the profile derived from the corresponding observational values, which has a uniform distribution from the bed to the water surface. With the rest of the data series, EDMTC estimates roughly higher partial values equal to the observational values for y*, as the RMSE error value is about 0.9–0.13, which is acceptable. Therefore, EDMTC with low average error values (MARE = 0.55 and MAE = 0.19) is generally highly accurate in predicting bank profiles and stable channel dimensions.

### 4.3. Uncertainty Analysis of the Proposed EDMTC and GEP Model

In this section, the uncertainty of EDMTC in predicting the bank profile shape based on entropy model ans also GEP model in predicting $\bar{S_{t}}$ of bank (according Equation (12)) is examined and the uncertainty indices are shown in Table 5. With the Uncertainty Wilson Score Method (UWSM) [10], ref. [19,75,76,77,78,79], the error of the $\bar{S_{t}}$ predicted by the GEP model and the y* values predicted by EDMTC is calculated and compared with the corresponding observation values. The error between estimated and observed values ($e_{i}$) and the corresponding the Mean Prediction Error (MPE or $\bar{e}$) and standard deviation ($S_{d}$) for error values calculated for data is obtained as Equations (21)–(23):

(21) $$e_{i}=x_{i}-y_{i},$$

(22) $$MPE=\bar{e}=\;\frac{1}{n}\sum_{i=1}^{n}e_{i},$$

(23) $$S_{d}=\sqrt{\sum_{i=1}^{n}\left(\frac{{\left(e_{i}-\bar{e}\right)}^{2}}{n-1}\right)},$$

where *n* is the sample size. With these indices, the *WUB* are calculated as Equation (24):

(24) $$WUB=\;\frac{1}{n^{0.5}}(I_{lt}\;S_{d}),$$

where $I_{lt}$ is the left-tailed inverse of the error distribution that represent the probability of error distrubution associated with the numebr of degree of freedom with which to characterize the distribution [76,80]. In the present paper, the probability of 0.05 error (95% Confidence Bound (CB)) with degree of freedom equals to *n* − 1 is considered in $I_{lt}$-value calculation [80]. Moreover, CB is the 95% quantile of the $I_{lt}$ distribution with 1 degree of freedom. In the following, CB can be defined. In this range, the *WUB* represents the upper and lower uncertainty bounds of CB respectively as Upper Bound (*UB*) and Lower Bound (*LB*). *UB* and *LB* can be calculated by $\bar{e}\pm \;WUB$. Moreover, the CB represents the mean value of error. Furthermore, ${\bar{d}}_{x}$ represents the average width of CB which is calculated as Equation (25). The lower average width of the CB associated with the lower values of $S_{d}$ and *WUB* provides the high certainty of model.

(25) $${\bar{d}}_{x}=\;\frac{1}{n}\sum_{i=1}^{n}\left(UB-LB\right)=\;\frac{1}{n}\;\sum_{i=1}^{n}\bar{e}\pm WUB,$$

The ideal certainty analysis is achieved when most of the estimated values are bracketed within the CB and also the narrowest width is achieved.

**Figure 5:**
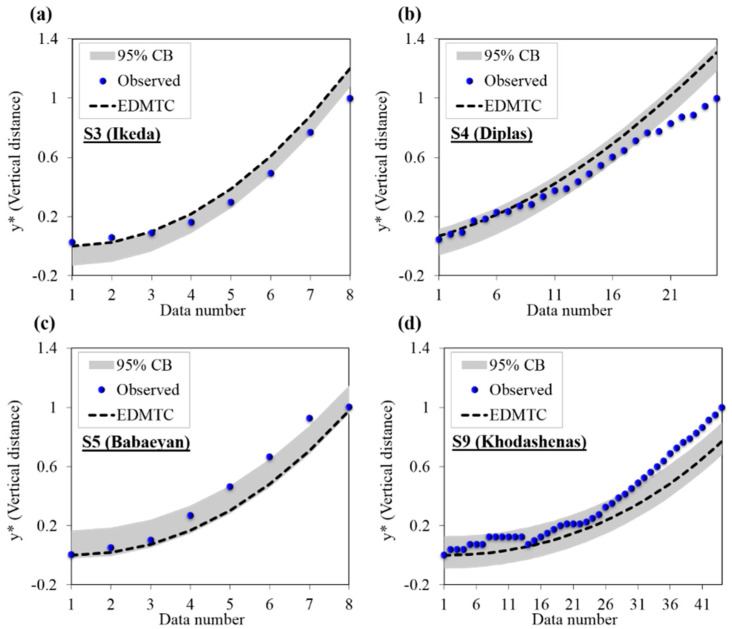

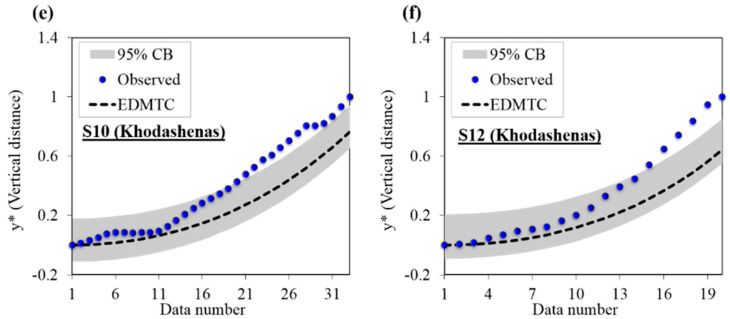
CB (95%) ranges for the observational values and values predicted by EDMTC for the vertical boundary elevation of stable channels based on different datasets of (**a**) Ikeda [20] (S3), (**b**) Diplas [21] (S4), (**c**) Babaeyan [7]-S5, (**d**) Khodashenas [68]-S9, (**e**) Khodashenas [68]-S10, and (**f**) Khodashenas [68]-S11.

Table 5 shows the MPE, CB, ${\bar{d}}_{x}$, and *WUB* for predicting the $S_{t}{}^{-}$ using the GEP model as well as the values of these indices for the EDMTC. Figure 5 displays the CB calculated using MPE for several observational data series (S3, S4, S5, S9, S10, and S12). In EDMTC, according Table 5, for all datasets, the low values of ${\bar{d}}_{x}$ (0.14), *WUB* (±0.04) and the low value of MPE (−0.14) represent the low uncertainty and high precision of proposed EDMTC in predicting y* values. It is clear that for almost each observational data series, 95% of predicted and observed values are within the CB range beside the narrow *WUB*. This represents the acceptable accuracy of the proposed models in predicting the vertical boundary elevation of stable channel profiles. According Table 5, in S3 [20] and S5 [7] data, almost all of the y* values predicted by EDMTC model are located within the one side of CB. Because, in these series of data, the more underestimation and overestimation performance of the EDMTC causes the almost high values of *WUB*. Morover, CB is calculated based on mean error values, therefore, the higher and lower predicted y* values than observed values are located in one side of CB. For the rest of the data, as more than 95% of the data are within this bound. According Table 5, the *WUB* in all test is low for EDMTC and for GEP model the *WUB* is 0.01. The low *WUB* and associate with the low ${\bar{d}}_{x}$ values provides a high certainty and precision of EDMTC for S3 [20] and S4 [21], and S5 [7]. While in S12 [68] the low values of *WUB* is associated with high $S_{d}$ values. The low values of $S_{d}$ and *WUB* in GEP model represents the high precision (low MPE value) and certainty of model simultaneously. Therefore, according to the explanations and results presented, it can be said that the proposed EDMTC and GEP has great certainty and their ability to predict the dimensions and stable bank profiles with high accuracy is assured. Therefore, the models proposed in this study can be used to predict channel dimensions in cases when there is little channel information given. Besides, the proposed model is capable of predicting the profile shape of stable channel banks when observational data for the bank profile shape is not available.

Finally, the proposed EDMTC can be used to determine the maximum value of y* as the maximum dimensionless depth at the channel center and the predicted free surface width. In this case, the channel dimensions can be obtained using the proposed model.

## 5. Conclusions

In the present study, the maximum entropy principle was employed to provide an equation to calculate the Lagrange multipliers. Accordingly, an equation was developed to predict the bank profile shape of threshold channels. The relation between (*δ*) ratio with the entropy parameter (*K*) and the hydraulic and geometric characteristics of channels was evaluated. Next, the EDMTC computational model for estimating the shape of banks profiles and the channel dimensions (*B* and *h_c_*) was designed based on the maximum entropy principle in combination with the GEP regression model for cases when only the *Q* and *d*_50_ are known as problem conditions. The results indicate that the entropy model is capable of predicting the bank profile shape trend with acceptable error values (MARE = 0.317, RMSE = 0.09) according to the experimental data in comparison with the Cao and Knight’s [28] model (MARE = 0.317, RMSE = 0.09). Therefore, the *λ* multiplier has a significant role in determining the transverse slope and consequently the vertical elevation of banks, and the physical meaning of *λ* is associated with the hydraulic parameters governing the problem. The EDMTC proposed in this study with *R*^2^ greater than 0.95 and MAE in the 0.076–0.436 range for different observational data series is able to predict the bank profile shape trend as well as the free water surface level in threshold channels. In addition, the uncertainty analysis of EDMTC demonstrated that more than 95% of predicted and observed data are within the CB with low *WUB*, and the model reliability is largely assured. The EDMTC computational model presented in this paper can be used widely to predict stable channel profiles when the given problem information only includes the *Q* and *d*_50_. This study was developed on Shannon entropy concept, it is suggested to improve the obtained results with other generalized entropies. It is further recommended that other equations provided by different researchers be used to estimate the free surface width of channels. Regression and AI models based on more field data also ought to be used to estimate the mean transverse slope of banks as well as other entropy model types to examine the accuracy of the model presented in this study.

## Figures and Tables

**Table 1:** Coefficients in the bank profile shape equation related to Vigilar and Diplas [11] (Equation (13)) for different values of *μ* and δ*_cr_ [11].

a_0_	a_1_	a_2_	a_3_	δ*_cr_
**μ = 0.4**				
1.0001	−0.0135	−0.0411	0	0.93
1.0004	−0.0236	−0.0412	0	0.935
1.0008	−0.0307	−0.0412	0	0.94
1.0009	−0.0342	−0.0413	0	0.945
**μ = 0.55**				
1.0003	−0.018	−0.0503	−0.0029	0.9
1.0006	−0.0299	−0.0527	−0.0027	0.905
1.0008	−0.0366	−0.0547	−0.0025	0.91
1.001	−0.0416	−0.0565	−0.0022	0.915
1.0011	−0.0463	−0.0586	−0.0019	0.921
**μ= 0.65**				
1.0006	−0.0278	−0.0543	−0.006	0.885
1.001	−0.0444	−0.06	−0.0054	0.895
1.0013	−0.0529	−0.0647	−0.0048	0.905
1.0041	−0.0556	−0.0665	−0.0045	0.909
**μ= 0.76**				
1.0009	−0.0365	−0.0544	−0.0105	0.87
1.0014	−0.0531	-0.061	−0.0101	0.88
1.0017	−0.0621	−0.0662	−0.0095	0.89
1.0018	−0.0662	−0.0701	−0.009	0.897
**μ= 0.84**				
1.0011	−0.0418	−0.0516	−0.0146	0.86
1.0016	−0.0594	−0.059	−0.0143	0.87
1.002	−0.0697	−0.0634	−0.0141	0.88
1.0021	−0.0742	−0.0708	−0.013	0.89
**μ= 1.0**				
1.0016	−0.0571	−0.0466	−0.0233	0.845
1.0022	−0.0738	−0.0531	−0.0237	0.855
1.0025	−0.0828	−0.0589	−0.0236	0.865
1.0028	−0.0884	−0.0656	−0.023	0.875
1.0028	−0.0892	−0.0683	−0.0226	0.878

**Table 2:** Summary of experimental characteristics for the data used in the present study.

Researchers	Runs. No.	No. of Series	*d*_50_ [mm]	Discharge (*Q*) [L/s]	Water Surface Half-Width (*B*/2) [cm]	Central Water Depth (*h_c_*) [cm]
Mikhailova et al. [65]	2	S1	0.2	65	112	10.4
		S2	0.2	69	132.5	14.4
Ikeda [20]	1	S3	1.3	16.28	24.8	3.54
Diplas [21]	1	S4	1.9	12.526	33	3.85
Babaeyan [7]	1	S5	1	2.5	52.6	2.63
Macky [66] (Field data)	1	S6	3.42	64.3	127	3.7
Hassanzadeh et al. [67]	2	S7	1.2	11.09	32	8.6
		S8	1.6	20.07	40.6	10.9
Khodashenas [68]	4	S9	0.53	6.2	21.7	8
		S10	0.53	2.57	16	6.3
		S11	0.53	2.18	17	6.12
		S12	0.53	1.157	9.5	3.7

**Table 3:** Assessment of the efficiency of developed entropy model (DEM) and CKM compared with different observational data series according to different error indices and *λ* values related to DEM in this paper.

	MARE		RMSE		Bias		R^2^		λ
Data Series	DEM	CKM	DEM	CKM	DEM	CKM	DEM	CKM	DEM
S1	0.254	1.95	0.103	1.31	−0.04	0.99	0.93	0.981	−5.56
S2	0.86	3.95	0.057	0.7	−0.036	0.47	0.98	0.988	−4.26
S3	0.228	0.47	0.037	0.141	0.022	0.116	0.99	0.981	−1.62
S4	0.15	0.11	0.053	0.08	−0.05	0.064	0.99	0.997	−1.75
S5	0.43	0.42	0.1	0.135	−0.08	0.114	0.99	0.988	2.11
S6	0.58	1.03	0.38	0.5	−0.31	0.45	0.96	0.957	1.5
S7	0.147	0.86	0.056	0.37	0.045	0.35	0.98	0.966	−2.46
S8	0.315	0.99	0.109	0.34	0.098	0.32	0.97	0.95	−2.2
S9	0.26	0.50	0.044	0.184	0.008	−0.148	0.98	0.989	1.72
S10	0.18	0.56	0.028	0.24	−0.01	−0.192	0.99	0.987	2.2
S11	0.23	0.46	0.05	0.14	0.03	−0.108	0.99	0.996	1.4
S12	0.17	0.47	0.05	0.22	0.03	−0.16	0.985	0.996	2.4
Averaged	0.317	0.981	0.08	0.363	−0.02	0.189	0.978	0.981	-

**Table 4:** Evaluation of the EDMTC proposed in the present study in estimating the dimensions of stable channels in comparison with several available observational data series.

Dataset	R^2^	MARE	RMSE	MAE	Bias
Ikeda [20] (S3)	0.995	0.357	0.098	0.078	0.064
Diplas [21] (S4)	0.991	0.186	0.132	0.097	0.094
Babaeyan [7] (One set) (S5)	0.961	0.400	0.124	0.095	−0.095
Macky [66] (S6)	0.942	0.568	0.556	0.381	0.380
Hassanzadeh et al. [67] (S7)	0.986	1.164	0.456	0.436	0.436
Hassanzadeh et al. [67] (S8)	0.981	1.146	0.380	0.364	0.364
Khodashenas [68] (S9)	0.992	0.426	0.127	0.109	−0.109
Khodashenas [68] (S10)	0.979	0.473	0.169	0.143	−0.143
Khodashenas [68] (S11)	0.994	0.361	0.096	0.076	−0.076
Khodashenas [68] (S12)	0.995	0.475	0.193	0.147	−0.147
Average	0.9816	0.5556	0.2331	0.1926	0.0768

**Table 5:** Uncertainty analysis for the Gene Expression Programming (GEP) model in $\bar{S_{t}}$ prediction according to Equation (12) and EDMTC.

Model	Datasets	Sample Number	$S_{d}$	*MPE*	*WUB*	${\bar{d}}_{x}$	*CB*
EDMTC	Ikeda [20] (S3)	8	0.08	−0.064	±0.07	0.065	−0.13 to 0.00
	Diplas [21] (S4)	25	0.09	−0.094	±0.04	0.09	−0.13 to −0.05
	Babaeyan [7] (S5)	8	0.08	0.095	±0.075	0.095	+0.02 to +0.17
	Khodashenas [68] (S9)	44	0.07	0.109	±0.02	0.11	+0.09 to +0.13
	Khodashenas [68] (S10)	33	0.09	0.143	±0.035	0.145	+0.11 to +0.18
	Khodashenas [68] (S12)	20	0.13	0.147	±0.06	0.15	+0.09 to +0.21
	All datasets	266	0.33	−0.14	±0.04	0.14	−0.18 to −0.10
GEP, Equation (12)	All datasets	20	0.02	-0.009	±0.01	±0.01	−0.02 to 0.00
